# Synthesis and Luminescence Properties of a Novel Green-Yellow-Emitting Phosphor BiOCl:Pr^3+^ for Blue-Light-Based w-LEDs

**DOI:** 10.3390/molecules24071296

**Published:** 2019-04-03

**Authors:** Qi Wang, Meiling Xie, Minghao Fang, Xiaowen Wu, Yan’gai Liu, Zhaohui Huang, Kai Xi, Xin Min

**Affiliations:** 1 Beijing Key Laboratory of Materials Utilization of Nonmetallic Minerals and Solid Wastes, National Laboratory of Mineral Materials, School of Materials Science and Technology, China University of Geosciences, Beijing 100083, China; wangqi-cugb@foxmail.com (Q.W.); xmling1014@126.com (M.X.); xwwu@cugb.edu.cn (X.W.); liuyang@cugb.edu.cn (Y.L.); huang118@cugb.edu.cn (Z.H.); 2 Department of Materials Science and Metallurgy, University of Cambridge, Cambridge CB3 0FS, UK; kx210@cam.ac.uk

**Keywords:** green-yellow phosphor, BiOCl:Pr^3+^, photoluminescence, blue-light-based w-LEDs

## Abstract

The development of white-light-emitting diodes (w-LEDs) makes it meaningful to develop novel high-performance phosphors excited by blue light. Herein, BiOCl:Pr^3+^ green-yellow phosphors were prepared via a high-temperature solid-state reaction method. The crystal structure, luminescent properties, lifetime, thermal quenching behavior, and quantum yield were studied in detail. The BiOCl:Pr^3+^ phosphors presented several emission peaks located in green and red regions, under excitation at 453 nm. The CIE coordinates could be tuned along with the changed doping concentration with fair luminescence efficiency. The results also indicated that the optimized doping concentration of Pr^3+^ ions was at x = 0.0075 because of the concentration quenching behavior resulting from an intense exchange effect. When the temperature reached 150 °C, the intensity of the emission peak at 495 nm could remain at 78% of that at room temperature. The activation energy of 0.20 eV also confirmed that the BiOCl:Pr^3+^ phosphor exhibited good thermal stability. All these results indicate that the prepared products have potential to be used as a high-performance green-yellow-light-emitting phosphor for blue-light-based w-LEDs.

## 1. Introduction

White-light-emitting diodes (w-LEDs) are potentially useful solid-state lighting devices because of their long service life, high luminous efficiency, energy saving, and environmental protection [1]. The widely used commercial w-LEDs are usually realized by combining blue light LED chips with YAG:Ce^3+^ yellow phosphors [2]. However, these commercial w-LEDs are not ideal due to their poor color rendering index, since the YAG:Ce^3+^ yellow phosphors lack the red-light emission peaks [3]. Many references have also been reported to improve their optical performance. The most common approaches are to add red-emitting phosphors [4] such as Mn^4+^-activated fluoride [5], oxide phosphors [6], and red-emitting CdSe/ZnS semiconductor quantum dots [7] to YAG:Ce^3+^ yellow-emitting phosphor. However, these approaches might result in the reabsorption among different phosphors, potentially reduce luminous efficiency, and limit their further application for w-LEDs [8]. Therefore, we think it is still of great significance to explore novel yellow phosphors with emission peaks in the green, yellow, and red regions under the excitation of blue light [9].

The Pr^3+^ ion, with a [Xe] 4f^2^ configuration, is one of the most special activators for phosphors due to its complex energy level scheme [10]. Through transition from different energy levels, the Pr^3+^ ion can emit light from visible to infrared light regions [11]. Thus, various phosphors doped with Pr^3+^ were prepared using different host materials, such as the Gd_2_O_2_S:Pr^3+^ [12], CaTiO_3_:Pr^3+^ [13], BaMoO_4_:Pr^3+^ [14], LaMgAl_11_O_19_:Pr^3+^ [15], and β-SiAlON:Pr^3+^ [16]. If it is possible to dope only the Pr^3+^ ion—which can emit green and red emission lights simultaneously—into a novel host material, then the pure white light would be achieved by the excitation of blue light chips. This would be of great potential for application in w-LEDs [17].

It is also known that the BiOX (X = Cl, Br, I) oxyhalide compounds have been widely used as catalysts in photocatalysis due to their strong intra-layer bonding force and weak interlayer van der Waals interaction [18]. These compounds usually consist of unique layered structures, which can efficiently separate the photogenerated electron–hole pairs through the internal electric fields and further improve the charge transfer from host to activation center. Thus, the doped rare-earth ions in BiOX oxyhalides host can absorb the activation more conveniently, and emit more intense emission peaks [19,20]. Moreover, the radius and charge of Bi^3+^ ion are like those of Pr^3+^ [21], suggesting that the Bi^3+^ ions could be substituted by Pr^3+^ ions in the BiOX lattice. All these studies further indicate that the compounds in BiOX oxyhalides family could be considered as host materials for Pr^3+^ doping. Among these oxyhalide-based phosphors, the rare-earth ion-doped BiOCl phosphors have been rarely prepared. Herein, the green-yellow-emitting phosphors BiOCl:Pr^3+^ were prepared for the first time. Their crystal structure, luminescent properties, lifetime, thermal quenching behavior, and quantum efficiency were studied in detail. Our investigation shows that the prepared products can be used as green-yellow phosphors for blue-light w-LEDs.

## 2. Experimental Procedure

### 2.1. Material Synthesis

Bi_1−x_OCl:xPr^3+^ (x = 0, 0.0025, 0.005, 0.0075, 0.01, 0.03, 0.05) phosphors were prepared by a high-temperature solid-state reaction method [22,23,24]. The stoichiometric ratio was calculated according to the following reaction equation:

(1−x)Bi_2_O_3_ + 2NH_4_Cl + xPr_2_O_3_ → 2Bi_1−x_OCl:xPr^3+^+ 2NH_3_ + H_2_O (1)

Bi_2_O_3_ (99.9%), NH_4_Cl (99.9%), and Pr_2_O_3_ (99.9%) were selected as raw materials. Because NH_4_Cl volatilizes at high temperature, 20 mol% excess of NH_4_Cl was needed to compensate for the loss of volatilization. All these chemicals were evenly mixed in an agate mortar for about 30 min. Afterwards, the mixed powders were put into an alumina crucible and heated in a muffle furnace at 540 °C for 1 h. After natural cooling to room temperature, Bi_1−x_OCl:xPr^3+^ phosphors were taken out and ground into powder for further measurement. In order to verify the potentiality of Bi_1−x_OCl:xPr^3+^ phosphors on w-LEDs, an w-LED device was fabricated, combining a blue GaN chip with the BiOCl:Pr^3+^ and self-made K_2_GeF_6_:Mn^4+^ phosphors with a mass ratio of 100:1. Then, the mixed phosphors were dispersed in epoxy resin and coated on a blue GaN chip with an intense emission peak at 460 nm. The coated chip was dried in an oven at 100 °C for 3 h, and finally the w-LED device was obtained.

### 2.2. Characterization Methods

The powder X-ray diffraction (XRD) patterns of the samples were recorded by X-ray powder diffraction (AXS D8 Advance, Bruker, Corporation, Karlsruhe, Germany). The unit cell crystal structure of BiOCl was plotted by the VESTA program. Microstructure morphology was observed and studied by a field emission scanning electron microscope (FESEM, Zeiss supra-55, Oberkochen, Germany). Photoluminescence (PL) emission spectra and excitation (PLE) spectra were characterized by a FL-4600 fluorescence spectrophotometer (Hitachi, Tokyo, Japan), using a 150 W Xe lamp as an excitation source. The operating voltage of the photomultiplier tube of the spectrophotometer was 400 V. Combining the same spectrophotometer with the self-made computer-controlled heating device, the PL spectra at different temperatures were tested. The decay behavior and lifetimes of PL were recorded by a time-resolved luminescence spectrometer (FS5, Edinburgh Instruments Ltd., Edinburgh, UK) combined with microsecond Xe flash and time-correlated single photon counter system. The quantum yield was characterized by an FLS920 fluorescence spectrophotometer (Edinburgh Instruments Ltd.) with an integrated sphere, and the absorption was measured with BaSO_4_ powder as reference.

## 3. Results and Discussion

### 3.1. Phase Composition and Crystal Structure

**Figure 1:**
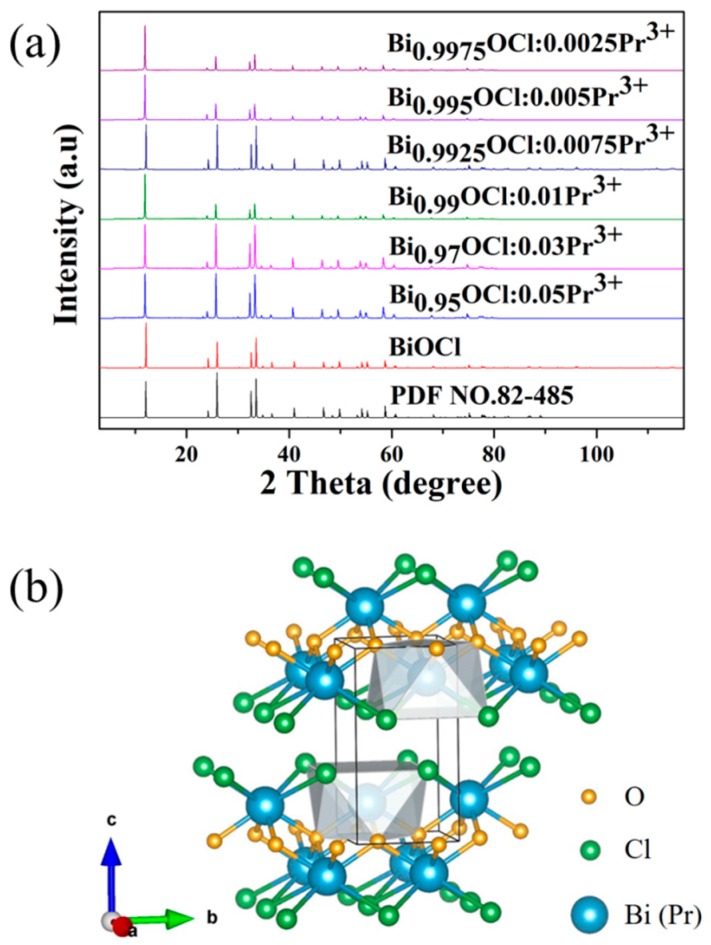
(**a**) XRD patterns of Bi_1−x_OCl:xPr^3+^ (x = 0, 0.0025, 0.005, 0.0075, 0.01, 0.03, 0.05) phosphors and the standard pattern (PDF NO. 82–485) of BiOCl; (**b**) the unit cell crystal structure of BiOCl.

The phase composition of the products was measured by XRD. Figure 1a shows the XRD patterns of Bi_1−x_OCl:xPr^3+^ (x = 0, 0.0025, 0.005, 0.0075, 0.01, 0.03, 0.05) samples and the standard pattern of BiOCl (PDF NO. 82-485). All the diffraction peaks of Bi_1−x_OCl:xPr^3+^ samples matched well with the BiOCl standard card. Almost no impurity peaks were presented. The addition of Pr^3+^ ions with different doping concentration had no significant effect on the crystalline structure of BiOCl host. Thus, it can be concluded that Bi_1−x_OCl:xPr^3+^ phosphors with stable structure can be easily prepared by this method. 

The crystal structure of BiOCl is presented in Figure 1b. It is clear that the BiOCl compound was crystallized in a tetragonal matlockite structure. The Bi^3+^ atom coordinated to a square antiprism with four O atoms on one side and four Cl atoms on the other side. The [Cl-Bi-O-Bi-Cl] layers were stacked together by van der Waals interactions between Cl atoms along the c-axis [25,26]. The interplanar lattice spacing between two Bi^3+^ ions in BiOCl layers was found to be 4.85 Å. Since the radius of Bi^3+^ is similar to that of Pr^3+^, Pr^3+^ ions can be easily doped into the lattice and successfully replace the position of Bi^3+^ ions without any structural changes. The XRD results and the discussion of crystal structure indicate that the Pr^3+^ ion could be doped into the BiOCl host at Bi^3+^ sites. 

### 3.2. PL properties of Bi_1−x_OCl:xPr^3+^ Phosphors

**Figure 2:**
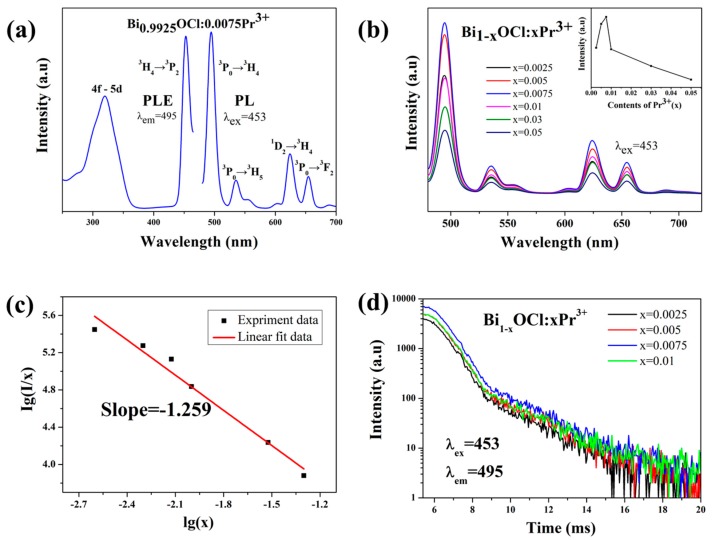
(**a**) Photoluminescence (PL) emission (λ_ex_ = 453 nm) spectra and excitation (PLE) (λ_em_ = 495 nm) spectra of a typical Bi_0.9925_OCl:0.0075Pr^3+^ sample at room temperature. (**b**) Emission spectra (λ_ex_ = 453 nm) and the relationship of the emission intensity on the content of Pr^3+^ of Bi_1−x_OCl:xPr^3+^ (x = 0.0025, 0.005, 0.0075, 0.01, 0.03, and 0.05). (**c**) Linear fitting experiment data of lg(*I*/*x*) versus lg(*x*) for the Bi_1−x_OCl:xPr^3+^ (x = 0.0025, 0.005, 0.0075, 0.01, 0.03, and 0.05) phosphors. (**d**) Decay curves of Bi_1−x_OCl:xPr^3+^ (x = 0.0025, 0.005, 0.0075, and 0.01) (λ_ex_ = 453 nm and λ_em_ = 495 nm) with different concentrations.

Figure 2a shows the PLE (λ_em_ = 495 nm) and PL (λ_ex_ = 453 nm) spectra of Bi_0.9925_OCl:0.0075Pr^3+^ phosphor at room temperature. By monitoring the emission at 495 nm, the excitation spectrum consisted of two peaks centered at 320 and 453 nm, respectively. The excitation peak at 453 nm is attributed to the electron transition from energy level ^3^H_4_ to ^3^P_2_. The absorption band from 280 to 350 nm might result from the 4f–5d characteristic transition absorption of Pr^3+^ ions [11]. In addition, the emission spectrum of Bi_0.9925_OCl:0.0075Pr^3+^ phosphor was composed of four peaks at 495, 535, 624, and 655 nm, under excitation at 453 nm. These emission peaks are attributed to the transitions of ^3^P_0_→^3^H_4_, ^3^P_0_→^3^H_5_, ^1^D_2_→^3^H_4_, and ^3^P_0_→^3^F_2_, respectively [27,28,29,30].

The emission spectra of Bi_1−x_OCl:xPr^3+^ (x = 0, 0.0025, 0.005, 0.0075, 0.01, 0.03, 0.05) with different doping concentrations are shown in Figure 2b. With different Pr^3+^ concentrations, the emission spectra were similar with each other. The emission peaks were at the same position, but their intensities were different. The inset of Figure 2b illustrates the dependence of emission intensity on Pr^3+^ concentration. With the increase of Pr^3+^ concentration, the emission intensity increased at first, reached the maximum when the doping concentration of Pr^3+^ ion was at x = 0.0075, then decreased with further increasing Pr^3+^ concentration. This phenomenon resulted from the concentration quenching effect. It is worth mentioning that the optimized doping concentration x = 0.0075 is actually a very low concentration, which means that the BiOCl:Pr^3+^ phosphor could emit light efficiently with fewer activators. This has also been seldom seen before, which could markedly reduce the cost of phosphors for w-LEDs.

There are many reasons for concentration quenching, including multipolar interaction or exchange interaction. The interaction types between the two types of incentives can be calculated by the following formula [31,32]:

*I*/*x* = *k*(1 + *β*(*x*)*^Q^*^/3^)^−1^ (2)

where *x* is the concentration of Pr^3+^ activator, and *k* and *β* are constants. *I*/*x* is the ratio of the emission intensity to the doping concentration of activator. The *Q* value determines the interaction type for the concentration quenching effect. When the values of *Q* are 3, 6, 8, and 10, the interaction could be classified as exchange interactions, dipole–dipole, dipole–quadrupole, and quadrupole–quadrupole interactions, respectively. Thus, the results of lg(*I*/*x*) and lg(*x*) are presented in Figure 2c to obtain the *Q* value. As shown in Figure 2c, the relationship between lg(*I*/*x*) and lg(*x*) was linear. After data fitting, *Q*/3 was found to be 1.259. Therefore, the calculated *Q* value is relatively close to the theoretical value of 3, which represents that the concentration quenching mechanism for the BiOCl:Pr^3+^ phosphors is exchange interaction.

The lifetime reflects the rate of the electron transiting from the maximum energy excited state to the ground state when the excitation light is removed. Figure 2d shows the decay curves of Bi_1−x_OCl:xPr^3+^ (x = 0, 0.0025, 0.005, 0.0075, 0.01) phosphors at 495 nm, when excited at 453 nm. The results show that the emission intensity decreased as time goes by. However, the decay curves remained stable with increasing Pr^3+^ concentration. All the decay curves of Bi_1−x_OCl:xPr^3+^ were well fitted by an exponential function [33]:

(3) $$I_{t}=Ae^{-\frac{t}{\tau }}+I_{0}$$

where *I_t_* and *I_0_* are emission intensities at time *t* and initial time, respectively, *A* is a constant, and *τ* is the lifetime for exponential components. According to the function, when the concentration *x* values were 0.0025, 0.005, 0.0075, and 0.01, the average lifetimes of Bi_1−x_OCl:xPr^3+^ phosphors were found to be 1.12, 1.13, 1.14, and 1.14 ms, respectively. These PL lifetimes of Bi_1−x_OCl:xPr^3+^ are relatively stable.

### 3.3. Thermal Stability of BiOCl:Pr^3+^ Phosphors

**Figure 3:**
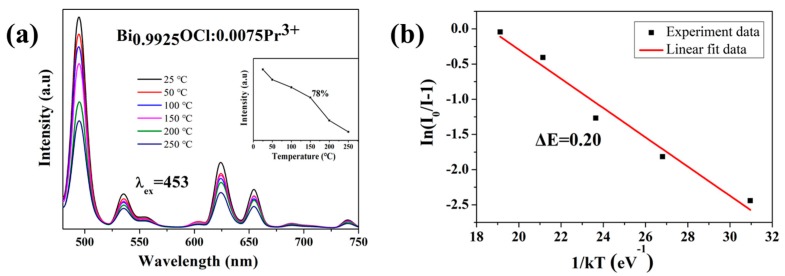
(**a**) PL spectra (λ_ex_ = 453 nm) of the Bi_0.9925_OCl:0.0075Pr^3+^ phosphor at different temperatures in the range of 25–250 °C. The inset in (**a**) shows the changes of the PL intensities of Bi_0.9925_OCl:0.0075Pr^3+^ with temperature. (**b**) Dependence of ln[*I*_0_/*I*(*T*)-1] on 1/*kT* for the Bi_0.9925_OCl:0.0075Pr^3+^ phosphor at the emission peaks of 495 nm.

The thermal stability of phosphors is an important factor for their application in w-LEDs [34]. Figure 3a shows the emission spectra of Bi_0.9925_OCl:0.0075Pr^3+^ phosphors at different temperatures, under excitation at 453 nm. As shown in Figure 3a, the PL intensity decreased with the increase of test temperatures. When the temperature reached 150 °C, the emission intensity of the peak at 495 nm remained at about 78% of the intensity at room temperature. Moreover, the central positions of emission peaks were not changed with increasing temperatures. To further investigate the temperature dependence of the luminescence property, the activation energy (Δ*E*) was calculated by the Arrhenius equation [35]:

(4) $$I\left(T\right)=\frac{I_{0}}{1+ce^{-\frac{\Delta E}{kT}}}$$

where *I_0_* is the emission intensity of phosphors at room temperature, *I*(*T*) is the intensity at different temperatures, *c* is a constant, and *k* is the Boltzmann constant (8.629 × 10^−5^ eV). According to the equation, Δ*E* could be calculated by Arrhenius fitting of the emission intensity of the Bi_0.9925_OCl:0.0075Pr^3+^ phosphor at different temperatures. Further, the smaller activation energy, the better thermal stability. As shown in Figure 3b, the relationship between ln [*I*_0_/*I*(*T*) – 1] and 1/*kT* was close to a straight line. The activation energy of Bi_0.9925_OCl:0.0075Pr^3+^ phosphor is calculated to be 0.20 eV, which is comparable to that of the phosphors reported in References [36,37]. All these results indicate that the prepared Bi_0.9925_OCl:0.0075Pr^3+^ phosphor has good thermal stability, which could have positive effects on its practical application.

### 3.4. Morphology and CIE Chromaticity Coordinates of Bi_0.9925_OCl:0.0075Pr^3+^ Phosphor

**Figure 4:**
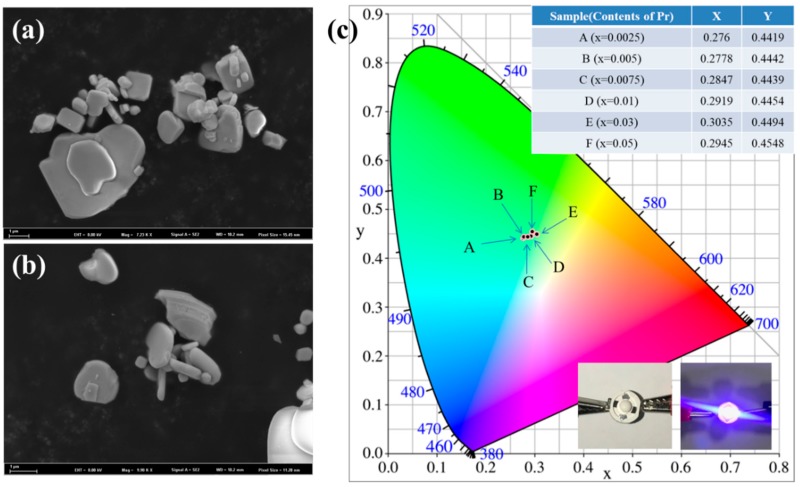
SEM micrographs of (**a**) BiOCl and (**b**) Bi_0.9925_OCl:0.0075Pr^3+^ phosphor. (**c**) The CIE chromaticity coordinate of the Bi_1−x_OCl:xPr^3+^ (x = 0.0025, 0.005, 0.0075, 0.01, 0.03, and 0.05) phosphors. The insets show the photographs of the fabricated white-light-emitting diode (w-LED) device before (left) and after (right) switching on the power.

The quality of a phosphor powder is related not only to its chemical and phase purity but also to the particle size and morphology [38]. Figure 4a,b are the FESEM morphologies of BiOCl and Bi_0.9925_OCl:0.0075Pr^3+^ samples, respectively. It can be seen that all these particles were in irregular oblate spheres. The particle size was uniform and about 1–3 μm. The average particle size was close to the optimal shape and size, which also had a positive impact on the luminescence performance.

As shown in Figure 4c, the CIE chromaticity coordinates are presented to evaluate the color purity of the Bi_1−x_OCl:xPr^3+^ (x = 0.0025, 0.005, 0.0075, 0.01, 0.03, and 0.05) phosphors. Under the excitation at 453 nm, the CIE coordinates of Bi_1−x_OCl:xPr^3+^ phosphor were calculated and dropped in the green-yellow light region. The CIE coordinate point gradually shifted to the yellow light region with increasing Pr^3+^ concentration. The PL quantum yields of the selected samples Bi_1−x_OCl:xPr^3+^ (x = 0.0025, 0.005, 0.0075, 0.01) under excitation at 453 nm were measured to be 35.4%, 30.8%, 24.9%, and 25.1%. Although the quantum efficiency values of BiOCl:Pr^3+^ phosphors were still lower than those of commercial phosphors, they could be increased through purification, surface coating treatment, or doping with other rare earth elements (e.g., Sm, Y, Li, and Bi) prior to further commercial applications [39,40,41]. Furthermore, the two insets show the photographs of the fabricated w-LED device before (left) and after (right) switching on the power. It can be seen that the fabricated w-LED device could emit white light, combining with a blue light chip and some red phosphors. All these results indicate the BiOCl:Pr^3+^ phosphors have good thermal stability, green-yellow spectrum, and light luminescence efficiency, which indicates that BiOCl:Pr^3+^ can be used as a green-yellow phosphor material and widely used in blue-light-based w-LEDs.

## 4. Conclusions

In conclusion, the BiOCl:Pr^3+^ phosphors were synthesized by a solid-state reaction method. Under excitation at 453 nm, the BiOCl:Pr^3+^ phosphors exhibited a green-yellow light with four emission peaks at 495, 535, 624, and 655 nm. These emission peaks are attributed to the ^3^P_0_→^3^H_4_, ^3^P_0_→^3^H_5_, ^1^D_2_→^3^H_4_, and ^3^P_0_→^3^F_2_ transitions, respectively. The optimized Bi_0.9925_OCl:0.0075Pr^3+^ phosphor was obtained with a CIE coordinate (0.2847, 0.4439). In addition, the Bi_0.9925_OCl:0.0075Pr^3+^ phosphor could also maintain a good thermal stability at high temperature. The intensity of emission peaks at 150 °C was about 78% of the initial intensity at room temperature. The quantum yield was measured to be 24.9%. The results show that BiOCl:Pr^3+^ phosphor is a green-yellow-light-emitting phosphor, which may be suitable for application in blue-light-based w-LEDs.
